# The Improvement of CRISPR-Cas9 System With Ubiquitin-Associated Domain Fusion for Efficient Plant Genome Editing

**DOI:** 10.3389/fpls.2020.00621

**Published:** 2020-05-21

**Authors:** Xuelian Zheng, Caiyan Qi, Lijia Yang, Quan Quan, Binglin Liu, Zhaohui Zhong, Xu Tang, Tingting Fan, Jianping Zhou, Yong Zhang

**Affiliations:** Department of Biotechnology, School of Life Sciences and Technology, Center for Informational Biology, University of Electronic Science and Technology of China, Chengdu, China

**Keywords:** CRISPR-Cas9, UBA domain, rice, genome editing efficiency, high efficiency

## Abstract

Genome editing technology represented by CRISPR-Cas9 had been widely used in many biological fields such as gene function analysis, gene therapy, and crop improvement. However, in the face of the complexity of the eukaryotic genome, the CRISPR-Cas9 genome editing tools have shown an unstable editing efficiency with large variability at different target sites. It was important to further improve the editing efficiency of the CRISPR-Cas9 system among the whole genome. In this study, based on the previous single transcription unit genome editing system (STU-SpCas9), using the ubiquitin-associated domain (UBA) to enhance the stability of Cas9 protein, we constructed three Cas9-UBA fusion systems (SpCas9-SD01, SpCas9-SD02, and SpCas9-SD03). Four different target sites of rice OsPDS, OsDEP1 and OsROC5 genes were chosen to evaluate the genome editing efficiency in rice protoplasts and stable transformed rice plants. The results showed that the fusion of UBA domains did not affect the cleavage mode of Cas9 protein, and effectively increase the editing efficiency of STU-SpCas9 at the target sites. This new CRISPR-Cas9-UBA system provided a new strategy and tool for improving the genome editing efficiency of CRISPR-Cas9 in plants.

## Introduction

The CRISPR-Cas9 system has been the most widely used genome editing technology for gene function analysis, gene therapy, and crop improvement in eukaryotic species because of its simple construction, high efficiency, and low cost (Li et al., 2013; Nekrasov et al., 2013; Shan et al., 2013; Liang et al., 2017; Lowder et al., 2018; Zhou et al., 2019). Numerous CRISPR-Cas9 tools have been developed to achieve targeted mutagenesis, base editing, precise editing by homology-directed repair (HDR) and transcriptional regulation in plants (Chen et al., 2019; Zhang et al., 2019). However, the CRISPR-Cas9 system still has some shortcomings such as off-target effects, ineffectiveness at some genomic sites, considerably variable editing efficiency, etc. The editing frequencies (insertion/deletion, indel) at different target sites are quite variable. For example in rice, the efficiency of some target sites was as high as 90–100%, while the others were less than 1% (Liu et al., 2017, 2019; Ding et al., 2019; Mao et al., 2019). Therefore, it is desirable to develop an ideal CRISPR-Cas9 system with sustained high activity at whole genome target sites.

The CRISPR-Cas9 editing efficiency is mainly dependent on two aspects: the genomic target sites and the CRISPR-Cas9 system itself. There have been some studies to alter the accessibility of genomic target site to improve editing efficiency *in vivo*, such as the proxy-CRISPR, CRISPR-chrom and Cas9-TV (Chen et al., 2017; Hille et al., 2018; Ding et al., 2019; Liu et al., 2019). There also have been many attempts to enhance the activity of CRISPR-Cas9 system to improve editing efficiency, which included expanding Cas9 nucleases and their variants, optimizing the structure of Cas9 for highly active protein, Cas9 codon optimization and improvement of sgRNA design, the dual Pol II promoter systems for high transcription level of Cas9 and sgRNAs, using strong promoters to enhance the transcription level of Cas9 gene in the expression system, etc. (Fauser et al., 2014; Cermak et al., 2015, 2017; Kleinstiver et al., 2015; Lowder et al., 2016; Tang et al., 2016; Casini et al., 2018; Hu et al., 2018; Chen et al., 2019; Mao et al., 2019; Zhang et al., 2019; Zhong et al., 2019). It is well known that the intracellular content of proteins is not only related to the expression level, but also to the degradation rate. Except for the traditional methods to improve the expression level through the transcription and translation of Cas9 protein, we consider whether there are other solutions to improve the intracellular content of Cas9 protein, such as inhibition of degradation.

Most proteins were degraded via the ubiquitin/26S proteasome pathway, which involved the participation of a large number of specific proteins and enzymes (Vierstra, 2009). A class of UBA (ubiquitin-associated) domains has been found in many proteins involved in protein degradation processes. In general, the UBA domains contained about 45 amino acid residues that were conserved from yeast to higher eukaryotes, the most conserved residues were generally non-polar, indicating that the UBA domain was unlikely to be directly involved in phosphorylation or ubiquitination (Hofmann and Bucher, 1996; Bertolaet et al., 2001). The yeast ubiquitin receptors Rad23, Dsk2, and Ddi1 delivered polyubiquitylated protein substrates to the proteasome for destruction. These receptors contained an N-terminal ubiquitin-like (UBL) domain that was recognized by the 26S proteasome and one or more C-terminal UBA domains that bound to substrates. However, these receptor proteins themselves escaped degradation and had long half-lives because of their C-terminal UBA domains. The C-terminal UBA domain of Rad23, Dsk2 and Ddi1 acted as an intrinsic stabilization signal that protected these receptors from proteasomal degradation by inhibiting multi-ubiquitin chain assemble or preventing the generation of initiation sites for degradation, which are required for proper engagement of the inherent unfolding machinery of the proteasome (Chen et al., 2001; Raasi and Pickart, 2003; Heessen et al., 2005; Raasi et al., 2005; Farmer et al., 2010; Heinen et al., 2011).

A few UBA chimeric protein studies have identified that the UBA domain can be used to enhance the stability of the target protein, prolong its half-life, and successfully improve the activity of the target protein. The UBA2 domain used to increase the stability of a destabilized GFP reporter protein in yeast (Heessen et al., 2005). The UBA1 and UBA2 domains of Arabidopsis ubiquitin receptor protein RAD23a functioned as a portable stable signal that extends the half-life of two unstable transcription factors HFR1 and PIF3 in Arabidopsis (Jang et al., 2012). The UBA domain of Arabidopsis DDI1 protein also increased the half-life of the unstable protein JAZ10.1 associated with jasmonic acid signaling (Jang et al., 2012). Based on these results, we have hypothesized that fusion modification of Cas9 protein with UBA domain might also increase the half-life of Cas9 protein and enhance Cas9 activity. Therefore, we tested this concept by fusing the Cas9 protein with three different Arabidopsis UBA domains (UBA1, UBA2, and UBA3) and developing the novel Cas9-UBA editing systems based on our previous reported STU-Cas9 system (Tang et al., 2016, 2019). The new Cas9-UBA genome editing systems effectively enhanced the activity of Cas9 protein and improve the editing efficiency of multiple target sites of OsPDS, OsDEP1, and OsROC5 genes in rice. These new systems can significantly improve the editing efficiency of target gene sites, and provides an alternative method for plant gene targeted mutagenesis and crop genome editing breeding.

## Results

### All Three Cas9-UBA Fusion Proteins Can Play Editing Activities in Rice Protoplasts

**FIGURE 1:**
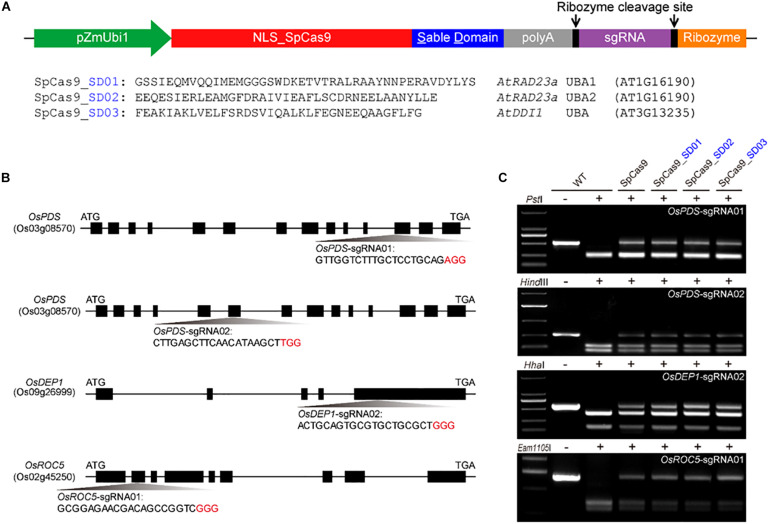
Comparison of three Cas9-UBA systems for rice genome editing. **(A)** Schematics of three Cas9-UBA expression systems: SpCas9-SD01 system, SpCas9-SD02 system, and STU- SpCas9-SD03 system. **(B)** Schematic diagram of genomic regions and four target sites of OsPDS, OsDEP1 and OsROC5 genes by STU-SpCas9 and three SpCas9-SD systems in rice. The PAM motif (NGG) is shown in red. **(C)** Mutagenesis as measured by loss of restriction enzyme sites due to targeted mutagenesis at four target sites.

**FIGURE 2:**
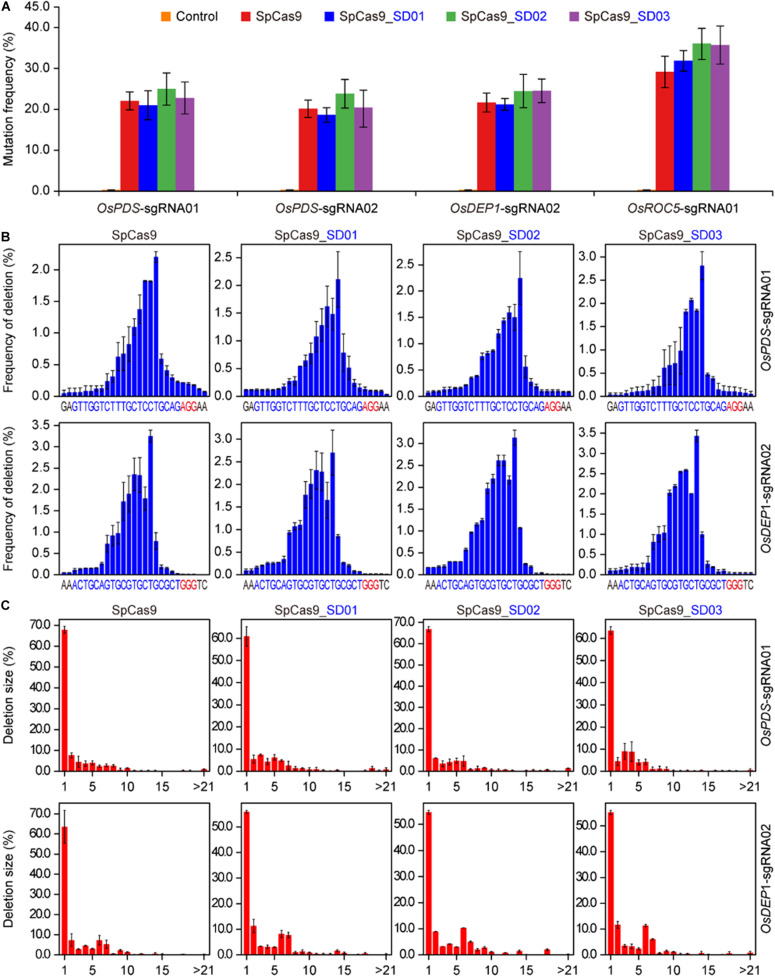
High-throughput sequencing analysis of three Cas9-UBA systems in rice cells. **(A)** Comparison of mutation frequencies of four STU-SpCas9 systems at four different target sites. **(B)** Comparison of positional deletion frequencies at OsPDS-sgRNA01 and OsDEP1-sgRNA02 target sites. The PAM sites are highlighted in red. **(C)** Comparison of deletion of different sizes at OsPDS-sgRNA01 and OsDEP1-sgRNA02 target sites. Each plant represents the same target site, while each column represents the same SpCas9 expression strategy. Error bars represent standard deviations of three biological replicates (*n* = 3).

To investigate whether Cas9 proteins fused with different UBA stable domains (SD) had editing activities, the three Cas9-UBA systems were used and compared to the STU-Cas9 (pGEL028) system (Figure 1A). With these four Cas9 systems (SpCas9, SpCas9-SD01, SpCas9-SD02, SpCas9-SD03), we targeted four sites (OsPDS-sgRNA01, OsPDS-sgRNA02, OsDEP1-sgRNA02, and OsROC5-sgRNA01, Figure 1B) in the rice genome. The resulting 16 constructs were used for transient transformation of rice protoplasts (Supplementary Table S2). All four Cas9 systems showed significant mutagenesis in four target sites resulting from error-prone NHEJ, as revealed by restriction fragment length polymorphism (RFLP) analysis (Figure 1C). The frequency of mutation was measured by high-throughput deep sequencing of PCR amplicons as a sum of insertion and deletions at the target sites. Mutation frequencies induced by the four Cas9 systems ranged from 20 to 30% across the four targets (Figure 2A). The STU-SpCas9 and three SpCas9-SD systems showed similar editing efficiencies and no significant differences were observed. This indicated that the fusion of different UBA SD domains does not affect the nuclease digestion activity of the Cas9 protein. The further analysis of NHEJ mutations in all samples showed no significant differences in the deletions profiles at target sites in the different Cas9-SD systems. Most of mutations produced by the four Cas9 systems were deletions, and the most frequent deletion positions were at 3 to 5 bp upstream of the PAM site (Figure 2B, Supplementary Figure S1A). The deletion majority ranged from 1 to 3 bp in size, and 1 bp deletions were the most predominant deletion type (Figure 2C, Supplementary Figure S1B). These results indicated that the fusion of the UBA domain does not influence the cleavage mode of Cas9 protein, and they were completely consistent with our previous report that the NHEJ repair outcomes are largely dictated by the sequence composition of the target sites but not the expression systems (Tang et al., 2016, 2019).

### Evaluation of STU-SpCas9 and Three SpCas9-SD Systems in Rice Transgenic Plants

**FIGURE 3:**
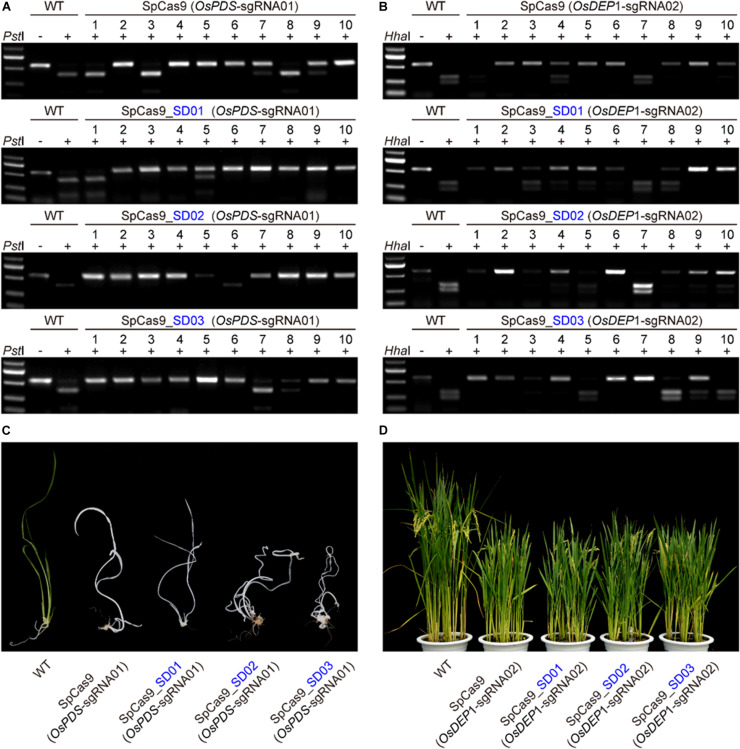
Evaluation of three Cas9-UBA systems editing frequency with rice stable transformation. **(A)** RFLP analysis of independent T0 plants of OsPDS-sgRNA01 site in different SpCas9-SD systems. **(B)** RFLP analysis of independent T0 plants of OsDEP1-sgRNA02 site in different SpCas9-SD systems. **(C)** Phenotype of wild type and targeted mutagenesis plants in different SpCas9-SD systems at the OsPDS-sgRNA01 target site. **(D)** Phenotype of wild type and targeted mutagenesis plants in different SpCas9-SD systems at the OsDEP1-sgRNA02 target site.

We further compared the editing efficiency of STU-SpCas9 and three SpCas9-SD systems with different UBA SD domains in stable transgenic rice plants. Eight constructs, respectively, targeting two rice genes (OsPDS-sgRNA01 and OsDEP1-sgRNA02) were transformed into rice calli mediated by Agrobacterium. Analysis of individual T0 transgenic plants from independent calli revealed that the two target sites had high efficiency mutation (Table 1). For OsPDS-sgRNA01 site constructs, more than 15 transgenic plants were obtained for RFLP analysis and genotyping with Sanger sequencing (Figure 3A, Supplementary Figure S2A). With the control STU-SpCas9 system, 12 out of 16 (75.0%) T0 plants were mutated and 9 plants (56.3%) carried bi-allelic mutations. For SpCas9-SD01 system, 12 out of 15 (80.0%) were mutated and 11 plants (73.3%) carried bi-allelic mutations. For SpCas9-SD02 system, 16 out of 18 (88.9%) were mutated of 13 plants (72.2%) carried biallelic mutations. For SpCas9-SD03 system, 14 out of 16 (87.5%) were mutated and 14 plants (87.5%) carried biallelic mutations. The albino phenotype among all biallelic mutants was observed since the *OsPDS* gene knockout (Figure 3C). The mutation rate and bi-allelic mutation efficiency of all three SpCas9-SD systems were higher than those of the STU-SpCas9 system. It suggested that UBA SD domains improved the editing efficiency of Cas9.

**TABLE 1:** Mutation rates of four Cas9 systems with OsPDS-sgRNA01 and OsDEP1-sgRNA02 in rice transgenic plants.

Constructs	Targeted locus	Tested T0 plants*	Mutated T0 plants (number; ratio*)	Biallelic mutation plants (number; ratio**)
SpCas9	*OsPDS*-sgRNA01	16	12; 75.0%	9; 56.3%
SpCas9_SD01		15	12; 80.0%	11; 73.3%
SpCas9-SD02		18	16; 88.9%	13; 72.2%
SpCas9_SD03		16	14; 87.5%	14; 87.5%
SpCas9	*OsDEP1-sgRNA02*	16	11; 68.8%	8; 50.0%
SpCas9_SD01		17	14; 82.4%	6; 35.3%
SpCas9-SD02		17	15; 88.2%	6; 35.3%
SpCas9_SD03		18	15; 83.3%	11; 61.1%

*All the tested T0 plants were transgenic positive plants confirmed by PCR. **Mutation rates in stable transgenic T0 plants. Each plant was genotyped by Sanger sequencing of PCR amplicons.

RFLP analysis and genotyping results similar to above were also observed in OsDEP1-sgRNA02 site constructs (Figure 3B, Supplementary Figure S2B). For the STU-SpCas9 control system, 11 out of 16 (68.8%) T0 plants were mutated and 8 plants (50.0%) carried bi-allelic mutations. For SpCas9-SD01 system, 14 out of 17 (82.4%) were mutated and 6 plants (35.3%) carried bi-allelic mutations. For SpCas9-SD02 system, 15 out of 17 (88.2%) were mutated of six plants (35.3%) carried biallelic mutations. For SpCas9-SD03 system, 15 out of 18 (83.3%) were mutated and 11 plants (61.1%) carried biallelic mutations (Table 1). Knockout of OsDEP1 results in semi-dwarf, panicle erectness, a reduced panicle length, an increased number of grains per panicle and a consequent increase in grain yield (Huang et al., 2009; Zhou et al., 2009). We compared the trait measures such as plant height, panicle length, grain number per panicle, 1000-grain weight, the grain length and width in the wildtype and all four Cas9 systems. All typical *DEP1* loss-of-function traits were observed in all biallelic mutants of the four Cas9 systems (Figure 3D, Supplementary Figure S3). But there was no significant difference among the phenotype of the STU-SpCas9 system and three STU-SpCas9 systems. These results indicated that the STU-SpCas9 systems are more efficient and robust than the STU-SpCas9 system for targeted mutagenesis in stable tranformation rice. Hence, the results from stable transgenic plants are consistent with those from rice protoplasts.

## Discussion

At present, CRISPR-Cas9-based genome editing has become the most widely used tool in various organisms. Numerous CRISPR-Cas9 tools and platforms have been developed, including the expanded nucleases and their variants, expression systems for multiplexing, precise genome editing with HDR or base editing, epigenome editing and transcriptome regulation (Liang et al., 2017; Lowder et al., 2018; Chen et al., 2019; Mao et al., 2019; Zhang et al., 2019; Zhou et al., 2019). Most of studies focus on increasing the editing efficiency of CRISPR-Cas9 system, especially the precise editing efficiency; expanding the genomic target ranges, achieving the sustained whole-genome editing; reducing off-target effect and improving specificity (Chen et al., 2019; Mao et al., 2019; Zhang et al., 2019). As mentioned before, improving the genome editing efficiency of CRISPR-Cas9 is mainly from two aspects: improving CRISPR-Cas9 system activity and improving the accessibility of genomic target sites. It is well known that protein expression level can be increased by enhancing the transcription and translation. Many previous studies are about how to increasing the expression level of Cas9 in cells. In this study we attempt to improve the genome editing efficiency of Cas9 from another perspective. The cleavage activity of Cas9 protein is closely related to its intracellular content, and the intracellular content of each protein is precisely regulated by cell. The balance of protein content is not only related to the expression level (input), but also to the degradation of the protein (output). It could be an effective strategy to increase the intracellular protein content by regulating the “input” and “output” of a protein at the same time. Most previous reports only focus on increasing Cas9 protein expression level (increasing input), but ignore the inhibition of Cas9 protein degradation (reducing output). Therefore, we consider to further enhance the stability of Cas9 protein and extend its half-life while maintaining high-level expression of Cas9 protein. This could increase the intracellular content of Cas9 protein, and improve the genome editing efficiency of Cas9 efficiently, stably and indiscriminately in the whole genome.

In a previous report, we demonstrated three promising STU-Cas9 systems for high-efficient plant genome editing, which were based on ribozyme, Csy4 and tRNA, respectively (Tang et al., 2016, 2019). STU-Cas9-RZ, STU-Cas9-Csy4, and STU-Cas9-tRNA systems all had similar editing efficiency in single and multiple gene target sites. Therefore, in this study, we constructed three STU-SpCas9-UBA systems (SpCas9-SD01, SpCas9-SD02, and SpCas9-SD03) based on STU-Cas9-RZ system to ensure the high-level expression of Cas9 protein (increased input). The UBA domain is a motif of about 40 amino acids, which competitively inhibited the degradation of the proteasome by binding to a substrate polyubiquitin chain (Raasi and Pickart, 2003; Raasi et al., 2005). The chimeric GFP reporter protein fused with the UBA domain at the C-terminus showed a significant increase in fluorescence levels in yeast (Heessen et al., 2005). A previous study in Arabidopsis showed that fusion of UBA1, UBA2, and UBA from AtRAD23a and AtDDI1 with endogenous transcription factors HFR1, PIF3 and intracellular jasmonic acid signaling protein JAZ10.1 significantly enhanced the stability of these proteins, and enhanced the functions of these proteins correspondingly (Jang et al., 2012). Therefore, we had chimeric three UBA domains to Cas9 protein separately to construct SpCas9-SD01, SpCas9-SD02, and SpCas9-SD03 fusion protein to enhance the stability of Cas9 and reduce its degradation at the same time (reduced output).

The original DNA sequences of UBA1, UBA2, and UBA domains were fused to Cas9 protein, respectively. The cleavage activity and genome editing efficiency of three fusion Cas9 proteins at four target sites were inspected in rice protoplasts. The results showed that the Cas9 proteins fused with the three original UBA domain sequences not only did not improve the genome editing efficiency of Cas9 protein, but decreased the editing activity at most of the target sites (data not shown). Considering the differences between rice and Arabidopsis, the rice gene expression system and protein degradation system were different from those of Arabidopsis. We had codon-optimized all three UBA domains (SD01, SD02, and SD03) according to the preference of rice codon usage. Three SpCas9-SD systems fused with codon-optimized SD domains showed similar cleavage activity in rice protoplast, which indicated that codon optimization was an effective method. The subsequent rice stable transformation results also showed that the codon-optimized SD domains successfully improved the genome editing efficiency of Cas9 protein at two endogenous gene target sites. Moreover, compared with SpCas9-SD01 and SpCas9-SD03, SpCas9-SD02 significantly improved the genome editing more efficiently at all target sites. This was consistent with previous reports that UBA2 has better stability and can more effectively extend protein half-life (Jang et al., 2012).

The UBA domain was a small motif that did not affect the function of chimeric proteins when increase the stability of targeted proteins. In our study, in addition to improving the editing efficiency, the C-terminal fused UBA domain did not affect the cleavage function of Cas9 protein. Three endogenous genes OsPDS, OsDEP1, and OsROC5 related to rice albino, semi-dwarf and panicle erectness, and curling leaf phenotype were selected. Genotyping analysis according to high-thought sequencing and Sanger sequencing in protoplasts and stable transformed plants showed that the cleavage mode of three SpCas9-SD systems were consistent with STU-Cas9 control in all four target sites. Almost all cleavages occurred 3, 4 nt near the PAM, and 1 bp indel mutation was the most common mutation type (Figure 2, Supplementary Figure S2). Our previous work revealed that the editing mode was not related to the expression system, mainly related to the characteristics around the target site (Tang et al., 2016, 2019), which was consistent in this study. On the other hand, phenotyping analysis of mutant plants in OsPDS-sgRNA01 and OsDEP1-sgRNA02 showed that three SpCas9-SD systems were not different from the STU-Cas9 control. At OsPDS-sgRNA01, all the mutant plants obtained through four Cas9 systems showed albino phenotype. And the phenotype of mutant plants at OsDEP1-sgRNA02 were semi-dwarf, panicle erectness, consistent with previous reports (Huang et al., 2009; Zhou et al., 2009). The fusion of UBA domain improved the editing efficiency of CRISPR-Cas9 system, which was generally considered to lead to the increase of off-target effect. However, in plant species, a large-scale whole genome sequencing was performed to detect cleavage of off-target sites by Cas9 nuclease in stably transgenic Arabidopsis, rice and cotton, revealing that Cas9 activity was highly specific with very low-level off-targeting. And that potential low-level off-target effects could be avoided by designing highly specific sgRNA, or be excluded by outcrossing to different varieties which is typical during commercial seed multiplication (Feng et al., 2014; Li et al., 2018; Tang et al., 2018; Chen et al., 2019). Thus, whether the SpCas9-SD systems will result in potential very low-level off-target should be considered and evaluated in follow-up study and agricultural application.

## Materials and Methods

### Plant Materials and Growth Condition

*Oryza sativa* L. japonica cultivar Nipponbare was used in this study. Plants were grown in soil in growth chambers at 28°C and 60% relative humidity under a long-day setting (16 h under the light and 8 h in the dark). For protoplast preparation, the sterilized seeds were placed in the 1/2 MS solid medium for 11 days in a dark chamber at 28°C. For the rice stable transformation, the sterilized seeds were placed in the N6-D solid medium to induce the callus for 7 days under light at 32°C.

### Construction of the Vectors

According to the UBA1 and UBA2 amino acid sequences of Arabidopsis AtRAD23a gene (GenBank: AT1G16190) and the UBA amino acid sequence of AtDDI1 gene (GenBank: AT3G13235), UBA-SD01, UBA-SD02, and UBA-SD03 DNA fragments were synthesized by rice codon preference optimization (Figure 1A and Supplementary Table S1).

We used Cas9 expression backbone vector pGEL028 (ZmUbi1-Cas9-NLS-polyA-RZ site-ccdB-gRNA-RZ) (Tang et al., 2016, 2019) for this study. To make CRISPR-Cas9-UBA gene edit system backbone vector pGEL113, pGEL115, pGEL116, the three Cas9-UBA fusion cassettes, SpCas9-SD01, SpCas9-SD02, SpCas9-SD03 were cloned into pGEL028 using fusion PCR and ligase, all primers are listed in Supplementary Table S1. For pGEL113 construction, DNA fragments 1 was obtained from PCR products using primers pTX72-Cas9-F, UBA1-A from template pGEL028, DNA fragments 2 was obtained from PCR products using primers UBA1-B and UBA1-C from UBA1 domain, and DNA fragments 3 was obtained from PCR products using primers UBA1-D and pTX72-ccdB-R template pGEL028, respectively. Fragments 1 and 2 were fused to make fragment 3 using primers pTX72-Cas9-F and pTX72-ccdB-R to get fragment 4. Finally, fragment 4 was cut by *Bsa*I and then cloned into *Bsa*I-digested pGEL028. The pGEL115, pGEL116 vectors were obtained through a similar method.

Four endogenous genes target site, OsPDS-sgRNA01, OsPDS-sgRNA02, OsDEP1-sgRNA02, and OsROC5-sgRNA01 were selected to construct expression vectors based on pGEL113, pGEL115, pGEL116 and pGEL028, respectively. sgRNAs were synthesized as duplexed oligonucleotides (Supplementary Table S1). Oligos were annealed and cloned into *Bsa*I linearized backbone vectors. All the vectors used in this study were provided as Supplementary Table S2.

### Rice Protoplast Transformation and Agrobacteria-Mediated Transformation

Rice protoplast transformation was performed as described previously (Zhang et al., 2013; Tang et al., 2016; Zhong et al., 2018, 2019), Each protoplast transformation experiment was performed in three biological replicates. Rice stable transformation was carried out as previously published protocol (Zheng et al., 2016; Zhong et al., 2018; Tang et al., 2019; Zhou et al., 2019).

### Detection of Targeted Gene Mutations

Restriction fragment length polymorphism (RFLP) and single-strand conformational polymorphism (SSCP) methods were used for mutation detection and analysis in protoplasts and T0 transgenic plants (Tang et al., 2016; Zheng et al., 2016). The genomic DNA was used for PCR with KOD FX DNA polymerase (TOYOBO) using primers OsPDS-F1/R1 for the OsPDS-sgRNA01 target site, primers OsPDS-F2/R2 for the OsPDS-sgRNA02 target site, OsDEP1-F/R for the OsDEP1-sgRNA02 target site, OsROC5-F/R for the OsROC5-sgRNA01 target site. OsPDS-sgRNA01 target site PCR amplicons were digested with *Pst*I, OsPDS-sgRNA02 target site PCR amplicons were digested with *Hin*dIII, OsDEP1-sgRNA02 target site PCR amplicons were digested with *Hha*I and OsROC5-sgRNA01 target site PCR amplicons were digested with *Eam*1105I. Editing in regenerated mutation T0 plants was confirmed by Sanger sequencing of PCR amplicons. The genotyping and the mutation ratio comparison in stable transgenic T0 plants were all based on sequencing results. All primers and restriction enzymes were listed in Supplementary Table S1.

### High-Throughput Sequencing Analysis

High-throughput sequencing analysis was carried out as published previously for detection and quantification of mutations for the protoplast DNA (Tang et al., 2017; Zhong et al., 2018). Genome regions of targeted sites were PCR-amplified using primers listed in Supplementary Table S1. Purified DNA samples were quantified and were sequenced using Illumina Hiseq 2,500 platform. Data processing was analyzed by CRISPRMatch (You et al., 2018). The mean averages and standard deviations of three biologically independent replicates were calculated.

### Measurement of Rice Yield Related Traits

The T1 generations originated from T0 mutation plants were used to test agronomic traits. The rice yield-related traits were measured according to previous method (Zhou et al., 2019). Three individual plants were used for data collection for each genotype. Each sample was tested for three times. The data were analyzed by Excel and SPSS.13 for calculation and significant differences analysis.

## Data Availability Statement

The sequencing data generated in this study can be found in NCBI using accession number PRJNA602039.

## Author Contributions

XZ and YZ conceived and designed the experiments. LY and XT generated all Vectors. CQ, LY, and ZZ performed rice protoplasts experiments and analyzed the HTS data. CQ, LY, BL, and QQ performed stable transgenic the rice experiments and analyzed the plants. CQ, LY, TF, and JZ performed the rice seeds analysis and other experiments. YZ and XZ analyzed the data and wrote the manuscript. All authors read and approved the final manuscript.

## Conflict of Interest

The authors declare that the research was conducted in the absence of any commercial or financial relationships that could be construed as a potential conflict of interest.

## Supplementary Material

FIGURE S1 Comprehensive analysis of three SpCas9-SD systems in rice protoplasts. **(A)** Comparison of positional deletion frequencies at two target sites OsPDS-sgRNA02 and OsROC5-sgRNA01. The PAM sites are highlighted in red. **(B)** Comparison of deletion sizes at two target sites. Each plant represents the same target site, while each column represents the same SpCas9 expression strategy. Error bars represent standard deviations of three biological replicates (*n* = 3).

FIGURE S2 Sanger sequencing reveals the genotype of mutant plants at OsPDS-sgRNA01 and OsDEP1-sgRNA02 sites in different SpCas9-SD systems. The PAM sequence is in red and the target sequence is in blue.

FIGURE S3 Targeted mutations of OsDEP1-sgRNA02 site led to diverse yield performance in different SpCas9-SD systems. **(A)** Comparison of panicle morphology in different SpCas9-SD systems. **(B)** Comparison of seed length and width in different SpCas9-SD systems. **(C)** Comparison of plant height (*n* = 7), panicle length, grain number per panicle and 1000-grain weight among all SpCas9-SD systems. Bar graphs show average mutation frequency from more than three biologically independent replicates with error bars representing standard deviations (*n* ≥ 3).

TABLE S1 Oligos used in this study.

TABLE S2 Constructs used in this study.
