# Supplementary material for: The Improvement of CRISPR-Cas9 System With Ubiquitin-Associated Domain Fusion for Efficient Plant Genome Editing
Source: Front Plant Sci. 2020 May 21;11:621. doi: 10.3389/fpls.2020.00621 (PMC7253670; doi:10.3389/fpls.2020.00621)
Supplement: FIGURE S1 — Comprehensive analysis of three SpCas9-SD systems in rice protoplasts. (A) Comparison of positional deletion frequencies at two target sites OsPDS-sgRNA02 and OsROC5-sgRNA01. The PAM sites are highlighted in red. (B) Comparison of deletion sizes at two target sites. Each plant represents the same target site, while each column represents the same SpCas9 expression strategy. Error bars represent standard deviations of three biological replicates (n = 3). [file Presentation_1.PPTX]

## Slide 1
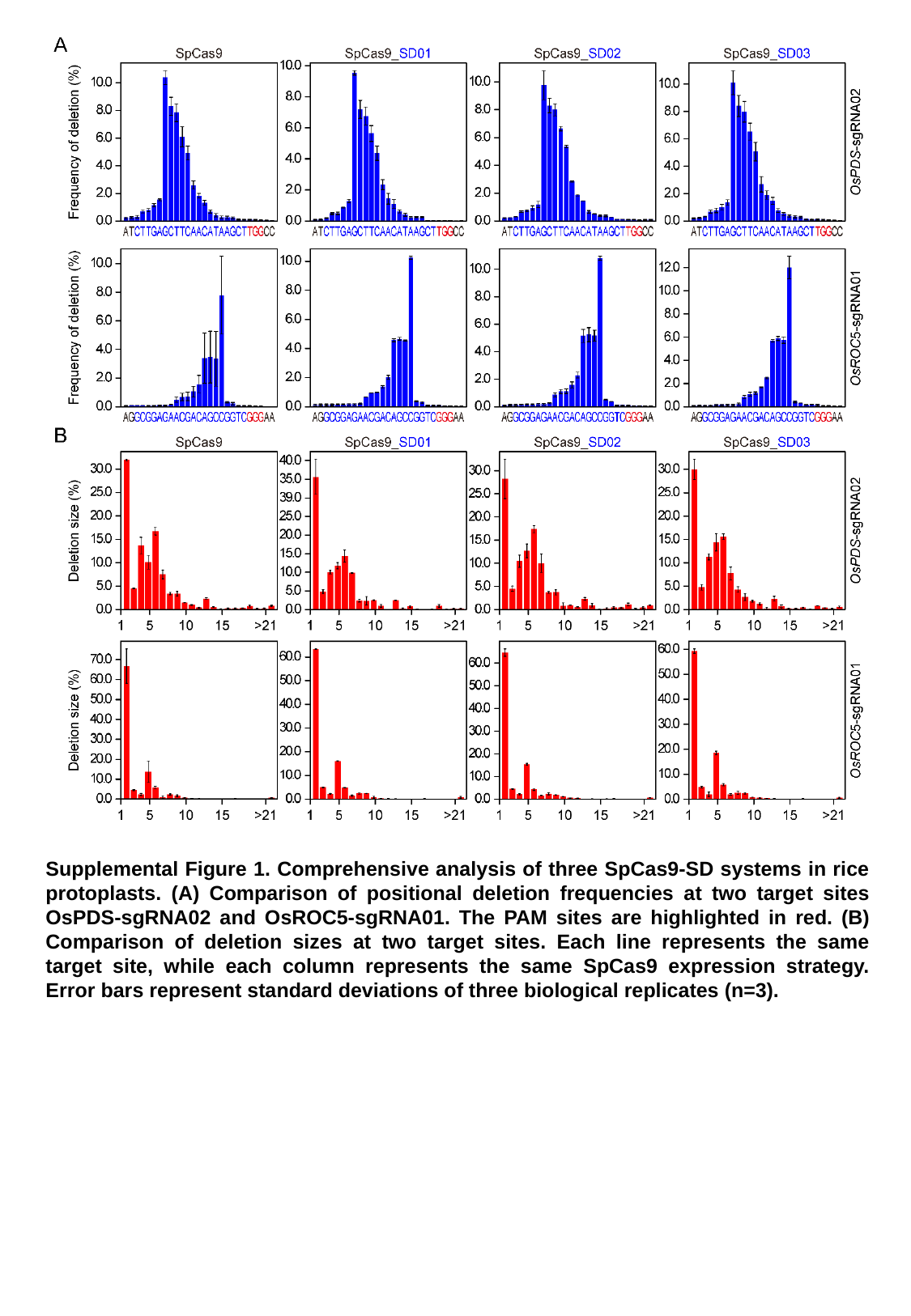

Supplemental Figure 1. Comprehensive analysis of three SpCas9-SD systems in rice protoplasts. (A) Comparison of positional deletion frequencies at two target sites OsPDS-sgRNA02 and OsROC5-sgRNA01. The PAM sites are highlighted in red. (B) Comparison of deletion sizes at two target sites. Each line represents the same target site, while each column represents the same SpCas9 expression strategy. Error bars represent standard deviations of three biological replicates (n=3).

## Slide 2
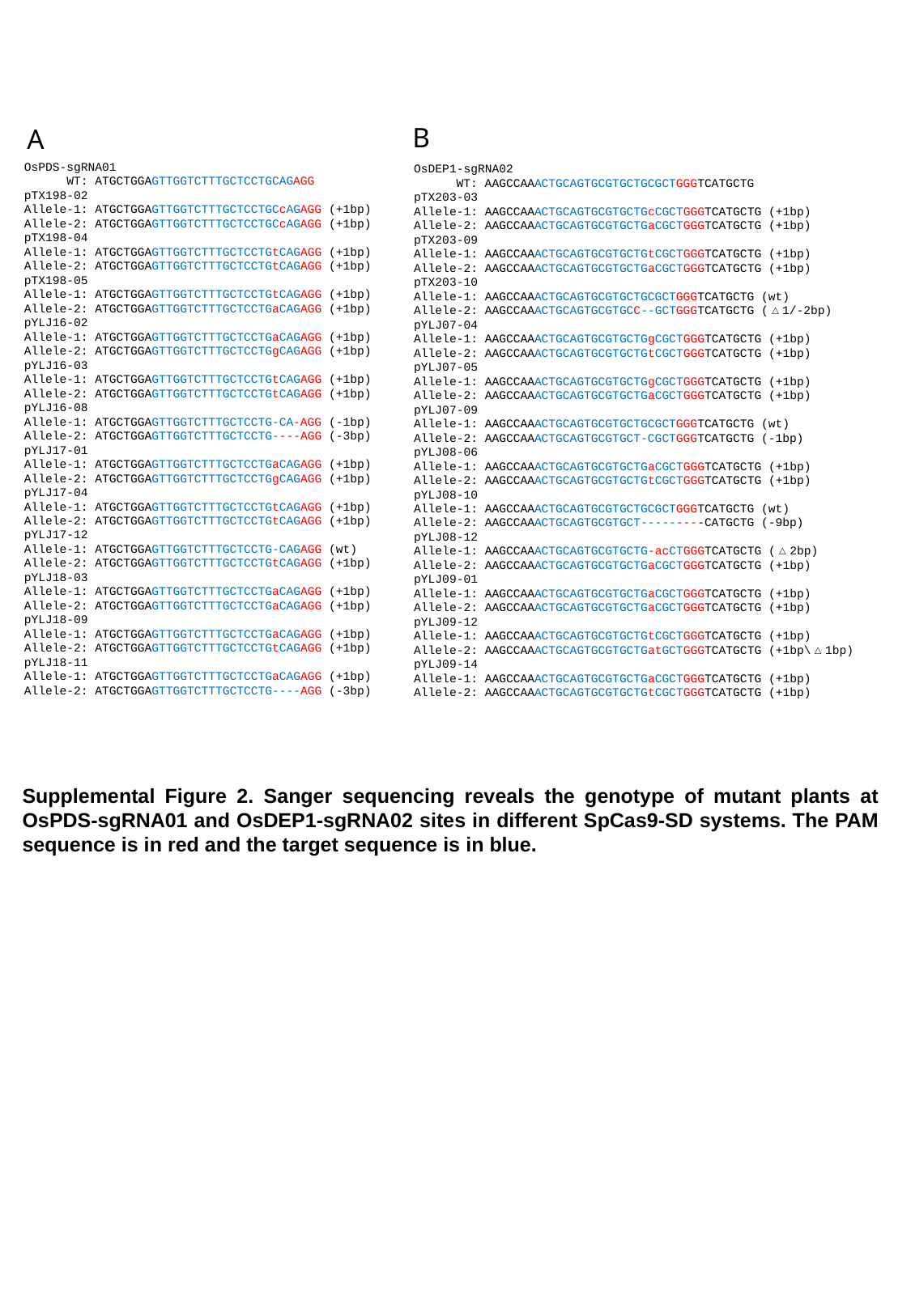

B
A
# OsPDS-sgRNA01 WT: ATGCTGGAGTTGGTCTTTGCTCCTGCAGAGG pTX198-02 Allele-1: ATGCTGGAGTTGGTCTTTGCTCCTGCcAGAGG (+1bp) Allele-2: ATGCTGGAGTTGGTCTTTGCTCCTGCcAGAGG (+1bp) pTX198-04 Allele-1: ATGCTGGAGTTGGTCTTTGCTCCTGtCAGAGG (+1bp) Allele-2: ATGCTGGAGTTGGTCTTTGCTCCTGtCAGAGG (+1bp) pTX198-05 Allele-1: ATGCTGGAGTTGGTCTTTGCTCCTGtCAGAGG (+1bp) Allele-2: ATGCTGGAGTTGGTCTTTGCTCCTGaCAGAGG (+1bp) pYLJ16-02 Allele-1: ATGCTGGAGTTGGTCTTTGCTCCTGaCAGAGG (+1bp) Allele-2: ATGCTGGAGTTGGTCTTTGCTCCTGgCAGAGG (+1bp) pYLJ16-03 Allele-1: ATGCTGGAGTTGGTCTTTGCTCCTGtCAGAGG (+1bp) Allele-2: ATGCTGGAGTTGGTCTTTGCTCCTGtCAGAGG (+1bp) pYLJ16-08 Allele-1: ATGCTGGAGTTGGTCTTTGCTCCTG-CA-AGG (-1bp) Allele-2: ATGCTGGAGTTGGTCTTTGCTCCTG----AGG (-3bp) pYLJ17-01 Allele-1: ATGCTGGAGTTGGTCTTTGCTCCTGaCAGAGG (+1bp) Allele-2: ATGCTGGAGTTGGTCTTTGCTCCTGgCAGAGG (+1bp) pYLJ17-04 Allele-1: ATGCTGGAGTTGGTCTTTGCTCCTGtCAGAGG (+1bp) Allele-2: ATGCTGGAGTTGGTCTTTGCTCCTGtCAGAGG (+1bp) pYLJ17-12 Allele-1: ATGCTGGAGTTGGTCTTTGCTCCTG-CAGAGG (wt) Allele-2: ATGCTGGAGTTGGTCTTTGCTCCTGtCAGAGG (+1bp) pYLJ18-03 Allele-1: ATGCTGGAGTTGGTCTTTGCTCCTGaCAGAGG (+1bp) Allele-2: ATGCTGGAGTTGGTCTTTGCTCCTGaCAGAGG (+1bp) pYLJ18-09 Allele-1: ATGCTGGAGTTGGTCTTTGCTCCTGaCAGAGG (+1bp) Allele-2: ATGCTGGAGTTGGTCTTTGCTCCTGtCAGAGG (+1bp) pYLJ18-11 Allele-1: ATGCTGGAGTTGGTCTTTGCTCCTGaCAGAGG (+1bp) Allele-2: ATGCTGGAGTTGGTCTTTGCTCCTG----AGG (-3bp)
OsDEP1-sgRNA02
 WT: AAGCCAAACTGCAGTGCGTGCTGCGCTGGGTCATGCTG
pTX203-03
Allele-1: AAGCCAAACTGCAGTGCGTGCTGcCGCTGGGTCATGCTG (+1bp)
Allele-2: AAGCCAAACTGCAGTGCGTGCTGaCGCTGGGTCATGCTG (+1bp)
pTX203-09
Allele-1: AAGCCAAACTGCAGTGCGTGCTGtCGCTGGGTCATGCTG (+1bp)
Allele-2: AAGCCAAACTGCAGTGCGTGCTGaCGCTGGGTCATGCTG (+1bp)
pTX203-10
Allele-1: AAGCCAAACTGCAGTGCGTGCTGCGCTGGGTCATGCTG (wt)
Allele-2: AAGCCAAACTGCAGTGCGTGCC--GCTGGGTCATGCTG (△1/-2bp)
pYLJ07-04
Allele-1: AAGCCAAACTGCAGTGCGTGCTGgCGCTGGGTCATGCTG (+1bp)
Allele-2: AAGCCAAACTGCAGTGCGTGCTGtCGCTGGGTCATGCTG (+1bp)
pYLJ07-05
Allele-1: AAGCCAAACTGCAGTGCGTGCTGgCGCTGGGTCATGCTG (+1bp)
Allele-2: AAGCCAAACTGCAGTGCGTGCTGaCGCTGGGTCATGCTG (+1bp)
pYLJ07-09
Allele-1: AAGCCAAACTGCAGTGCGTGCTGCGCTGGGTCATGCTG (wt)
Allele-2: AAGCCAAACTGCAGTGCGTGCT-CGCTGGGTCATGCTG (-1bp)
pYLJ08-06
Allele-1: AAGCCAAACTGCAGTGCGTGCTGaCGCTGGGTCATGCTG (+1bp)
Allele-2: AAGCCAAACTGCAGTGCGTGCTGtCGCTGGGTCATGCTG (+1bp)
pYLJ08-10
Allele-1: AAGCCAAACTGCAGTGCGTGCTGCGCTGGGTCATGCTG (wt)
Allele-2: AAGCCAAACTGCAGTGCGTGCT---------CATGCTG (-9bp)
pYLJ08-12
Allele-1: AAGCCAAACTGCAGTGCGTGCTG-acCTGGGTCATGCTG (△2bp)
Allele-2: AAGCCAAACTGCAGTGCGTGCTGaCGCTGGGTCATGCTG (+1bp)
pYLJ09-01
Allele-1: AAGCCAAACTGCAGTGCGTGCTGaCGCTGGGTCATGCTG (+1bp)
Allele-2: AAGCCAAACTGCAGTGCGTGCTGaCGCTGGGTCATGCTG (+1bp)
pYLJ09-12
Allele-1: AAGCCAAACTGCAGTGCGTGCTGtCGCTGGGTCATGCTG (+1bp)
Allele-2: AAGCCAAACTGCAGTGCGTGCTGatGCTGGGTCATGCTG (+1bp\△1bp)
pYLJ09-14
Allele-1: AAGCCAAACTGCAGTGCGTGCTGaCGCTGGGTCATGCTG (+1bp)
Allele-2: AAGCCAAACTGCAGTGCGTGCTGtCGCTGGGTCATGCTG (+1bp)
Supplemental Figure 2. Sanger sequencing reveals the genotype of mutant plants at OsPDS-sgRNA01 and OsDEP1-sgRNA02 sites in different SpCas9-SD systems. The PAM sequence is in red and the target sequence is in blue.

## Slide 3
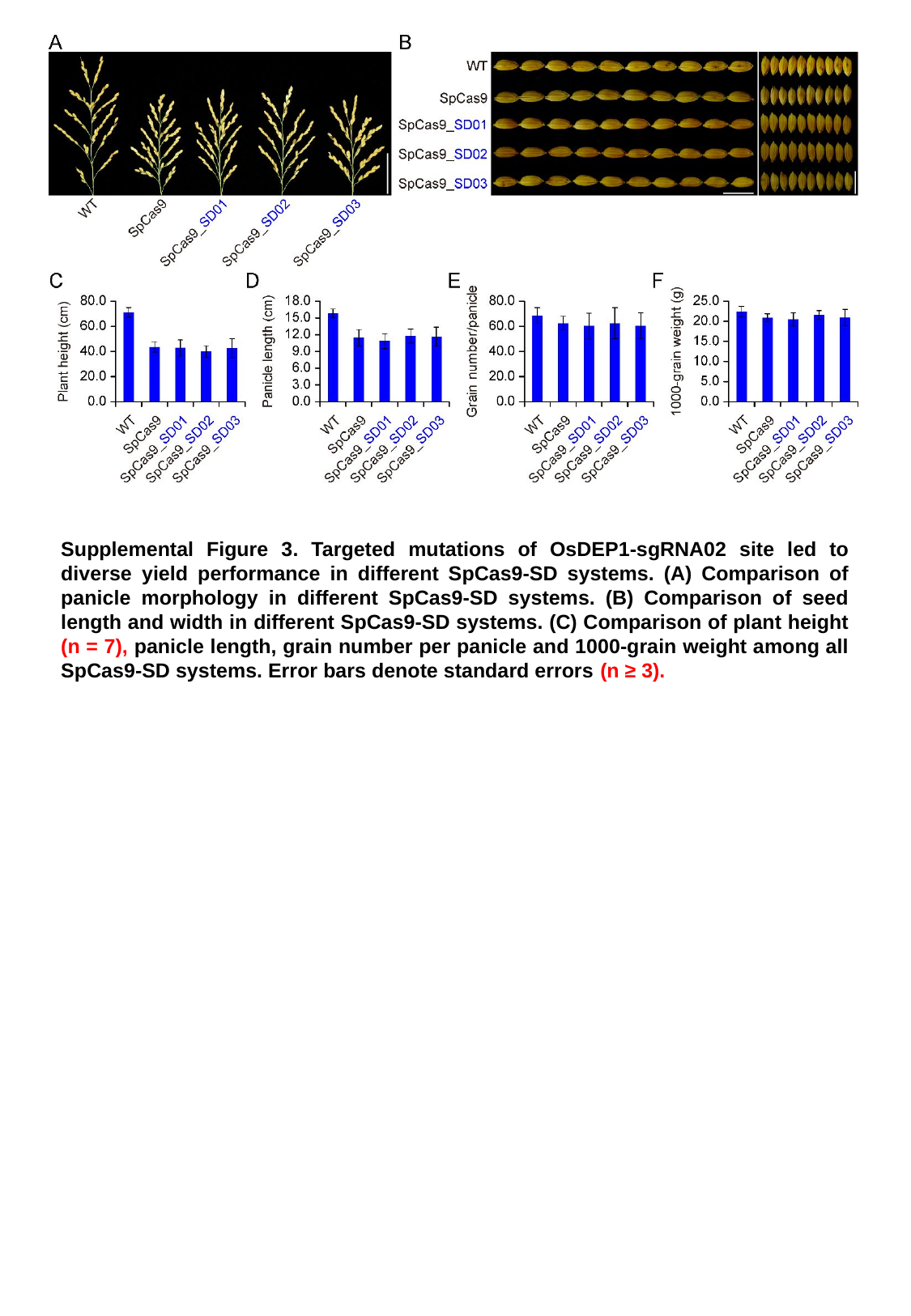

Supplemental Figure 3. Targeted mutations of OsDEP1-sgRNA02 site led to diverse yield performance in different SpCas9-SD systems. (A) Comparison of panicle morphology in different SpCas9-SD systems. (B) Comparison of seed length and width in different SpCas9-SD systems. (C) Comparison of plant height (n = 7), panicle length, grain number per panicle and 1000-grain weight among all SpCas9-SD systems. Error bars denote standard errors (n ≥ 3).
